# Ghrelin Derangements in Idiopathic Dilated Cardiomyopathy: Impact of Myocardial Disease Duration and Left Ventricular Ejection Fraction

**DOI:** 10.3390/jcm8081152

**Published:** 2019-08-01

**Authors:** Aneta Aleksova, Antonio Paolo Beltrami, Elisa Bevilacqua, Laura Padoan, Daniela Santon, Federico Biondi, Giulia Barbati, Elisabetta Stenner, Gianluca Gortan Cappellari, Rocco Barazzoni, Fabiana Ziberna, Donna R Zwas, Yosefa Avraham, Piergiuseppe Agostoni, Tarcisio Not, Ugolino Livi, Gianfranco Sinagra

**Affiliations:** 1 Cardiovascular Department, Azienda Sanitaria Universitaria Integrata di Trieste and Department of Medical Surgical and Health Sciences, University of Trieste, 34149 Trieste, Italy; 2 Department of Medicine (DAME), University of Udine, 33100 Udine, Italy; 3 Sport and Exercise Medicine Division, Department of Medicine, University of Padova, 35122 Padova, Italy; 4 Biostatistics Unit, Department of Medical Surgical and Health Sciences, University of Trieste, 34149 Trieste, Italy; 5 Unique Laboratory of Azienda Sanitaria Universitaria Integrata di Trieste, Burlo, Gorizia and Monfalcone, Azienda Sanitaria Universitaria di Trieste, 34125 Trieste, Italy; 6 Unit of Clinica Medica Generale e Terapia Medica, Department of Medical Surgical and Health Sciences, University of Trieste, 34149 Trieste, Italy; 7 Institute for Maternal and Child Health–IRCCS “Burlo Garofolo”, 34137 Trieste, Italy; 8 Linda Joy Pollin Cardiovascular Wellness Center for Women, Heart Institute, Hadassah University Medical Center, Jerusalem, Israel; 9 Department of Human Nutrition and Metabolism, School of Public Health Medical Faculty, Jerusalem 91120, Israel; 10 Centro Cardiologico Monzino, IRCCS, Department of Clinical Sciences, 20138 Milan, Italy; 11 Department of Clinical Sciences and Community Health, University of Milan, 20122 Milan, Italy; 12 Institute for Maternal and Child Health–IRCCS “Burlo Garofolo” Trieste and University of Trieste, 34137 Trieste, Italy; 13 Department of Medicine (DAME), University of Udine, and Cardiothoracic Department, University-Hospital Santa Maria della Misericordia, 33100 Udine, Italy

**Keywords:** idiopathic dilated cardiomyopathy, heart failure, ghrelin, GHS-receptor, cardiomyopathies, outcome, biomarkers

## Abstract

Background: Ghrelin may exert positive effects on cardiac structure and function in heart failure (HF) patients. Methods: We assessed ghrelin levels in 266 dilated cardiomyopathy (DCM) patients and in 200 age, gender and body mass index (BMI) matched controls. Further, we evaluated the expression of ghrelin and growth hormone secretagogue-receptor (GHSR) in the myocardium of 41 DCM patients and in 11 controls. Results: DCM patients had significantly lower levels of total, acylated and unacylated ghrelin when compared to controls (*p* < 0.05 for all). In controls, we observed a negative correlation of ghrelin with age, male gender and BMI. These correlations were lost in the DCM group, except for male gender. Total ghrelin was higher in patients with more recent diagnosis when compared to patients with longer duration of the DCM (*p* = 0.033). Further, total ghrelin was higher in patients with lower left ventricular systolic function (<40% LVEF, vs. 40% ≤ LVEF < 49% vs. LVEF ≥ 50%: 480.8, vs. 429.7, vs. 329.5 pg/mL, respectively, *p* = 0.05). Ghrelin prepropeptide was expressed more in DCM patients than in controls (*p* = 0.0293) while GHSR was expressed less in DCM patients (*p* < 0.001). Furthermore, ghrelin showed an inverse correlation with its receptor (ρ = −0.406, *p* = 0.009), and this receptor showed a significant inverse correlation with Interleukin-1β (ρ = −0.422, *p* = 0.0103). Conclusion: DCM duration and severity are accompanied by alterations in the ghrelin–GHSR system.

## 1. Introduction

Idiopathic dilated cardiomyopathy (DCM) is a progressive heart muscle disease, defined by the presence of left ventricular or biventricular dilatation and dysfunction [1], whose most common first manifestation is heart failure (HF). Patients usually show an initial improvement, within the first 24 months of treatment, a subsequent stabilization, lasting several years, and then a progressive worsening [2]. DCM affects young adults whose life expectancy and quality is greatly diminished despite available therapies [3]. Typical histological findings include cytological anomalies, loss of cardiomyocytes and interstitial fibrosis [4]. 

Based on current studies, it appears that ghrelin [5] may attenuate alterations in cardiac structure and function in patients with HF. Ghrelin is a gastric peptide, synthesized from a gene encoding for a prepropeptide that is subsequently cleaved to generate both ghrelin and obestatin. These peptides exert opposite effects on food intake, where the first is orexigenic, while the second is anorexigenic [6]. Both hormones are also expressed in the heart, though at a much lower level than in the gastrointestinal tract, where they can protect cardiomyocytes against apoptosis [7], inhibit myocardial fibrosis [8], activate the NO/Protein Kinase G pathway [9] and exert anti-inflammatory activity [10]. Ghrelin can also modulate the activity of the cardiac autonomic nervous system, improve cardiac contractility, and cardiomyocyte metabolism [11]. Systematic review and meta-analysis of animal HF studies of any cause suggests that ghrelin may lower the risk of mortality and improve cardiovascular outcomes [5]. Also, in a DCM animal model, ghrelin administration significantly improved the life expectancy compared with controls [12].

To date, only a few small studies investigated the role of ghrelin/obestatin in patients with DCM. Further, ghrelin levels in DCM patients with different HF duration, different left ventricular function and distinct New York Heart Association (NYHA) classes has not yet been investigated. 

Accordingly, the present study was designed to test ghrelin levels, in a large cohort of patients with DCM and long-term follow-up, and to investigate whether levels differ according to myocardial disease duration, NYHA class and left ventricular ejection fraction (LVEF). Furthermore, we aimed to compare ghrelin of DCM patients with age-, gender- and body mass index (BMI)-matched healthy controls. Last, we evaluated the expression of ghrelin prepropeptide, ghrelin receptor (i.e., the so-called growth hormone secretagogue-receptor, GHSR), and interleukin-1β (IL-1β) in the myocardium of patients with end-stage DCM and in controls.

## 2. Materials and Methods

### 2.1. Study Subjects

#### 2.1.1. Patients

A cohort of 266 patients affected by DCM referred to the Cardiology of Trieste (Trieste, Italy) and to the Centro Cardiologico Monzino (Milano, Italy) was prospectively enrolled between 01 January 2002 and 11 November 2016. The diagnosis of DCM was made according to the World Health Organization criteria [1]. Clinical and instrumental data were collected at the moment of enrolment and during each follow-up visit. Patients had scheduled visits at 6, 12 and 24 months, and every two years thereafter, or more frequently on the base of specific clinical needs. An accurate clinical-instrumental assessment of first-degree relatives of the proband is performed since it has fundamental relevance early recognition of asymptomatic affected relatives [13].

The cohort of patients was compared with a group of 200 healthy individuals comparable for age, gender and body mass index from Studio Epidemiologico MoMa, Friuli Region (Trieste, Italy). 

BMI was calculated as weight in kilograms divided by the square of height in meters. Standard echocardiographic evaluation was performed in all patients [14]. Total mortality or urgent heart transplantation were considered as endpoints. The indication for heart transplantation was status I—considering patients with refractory heart failure with necessity of inotropic treatment, mechanical circulatory support or both. Information regarding the endpoints was obtained from the patient, or from the registries of death of the communities of residence. The end of the follow-up was set at 01 August 2017 for censored patients or the date of death or heart transplantation of the patient. This study was performed in accordance with the guidelines set by the Declaration of Helsinki and was approved by the institutional ethics committee (ref. 43/2009-211/2014/Em). Written informed consent was obtained from all patients.

#### 2.1.2. Histological Analyses

Histological analyses were conducted on samples from 41 patients affected by DCM, who had undergone heart transplantation at Cardiac Surgery of Udine Hospital. The study, conducted in accordance with the Declaration of Helsinki, was approved by the Internal Review Board of the University of Udine (29 August 2017; ref. 10/IRB DAME_BELTRAMI_17). We used 11 heart samples from patients that died for causes other than cardiovascular disease as a comparison group.

### 2.2. Biomarkers Assessment and Histological Analyses

#### 2.2.1. Samples Processing

Venous blood samples for biomarker determination were collected in K_2_EDTA plasma tubes at study entry. Blood samples were kept chilled and then centrifuged at 2500 g for 10 min at 4 °C. Plasma was aliquoted in order to be stored at −80 °C until analysis was performed.

#### 2.2.2. Measurement of Plasma Biomarkers Levels

All plasma biomarker concentrations were determined by commercial enzyme-linked immunosorbent assay kits (ELISA) with specific monoclonal antibodies pre-coated onto a microplate and were performed according to manufacturer’s instructions. Optical density was measured using a microplate reader with different wave lengths (Multiskan FC, Thermo Scientific Oy, Vantaa, Finland).

Total and acylated ghrelin were measured with Human Ghrelin Active ELISA Kit and Human Ghrelin Total ELISA Kit (EMD Millipore Corporation, Billerica, MA, USA). Assay sensitivities were 20 pg/mL and 50 pg/mL respectively using a 20 μL sample size; furthermore, theoretical minimal identifiable concentration of acylated ghrelin with the Human Ghrelin Active ELISA Kit is 15 pg/mL. Levels of unacylated ghrelin were estimated by subtracting measured acylated ghrelin levels from total ghrelin levels. Quantikine HS ELISA kits (R&D Systems, Minneapolis, MN, USA) were used to determine plasma levels of IL-1β (Quantikine HS Human IL-1β ELISA), IL-6 (Human IL-6 Quantikine HS ELISA) and IL-10 (Human IL-10 Quantikine HS ELISA). IL-1β minimum detectable dose reported from producer was 0.057 pg/mL, while IL-6 and IL-10 assay’s sensitivity were 0.11 pg/mL and 3.9 pg/mL respectively. Tumor necrosis factor alpha (TNF-α) detection was done with Human TNF-α ELISA Kit (Life Technologies, Frederick, MD, USA) with an analytical sensitivity of 1.7 pg/mL. Brain natriuretic peptide (BNP) plasma concentration was analyzed with competitive enzyme immunoassay RayBio® Human/Mouse/Rat BNP Enzyme Immunoassay (RayBiotech, Norcross, GA, USA) with a detection threshold of 1.66 pg/mL. Human Galectin-3 Quantikine ELISA kit (R&D Systems, Minneapolis, MN, USA) was used to measure Galectin-3 plasma levels. The minimum detection dose referred was 0.016 ng/mL. Plasma levels of soluble suppressor of tumorigenicity-2 (sST2) were measured with ELISA Sandwich Presage® ST2 Assay (Critical Diagnostics, San Diego, CA, USA), which minimum detectable dose was 1.8 ng/mL for a 20 μL sample size.

### 2.3. Tissue Sampling and Immunohistochemistry Stains

Explanted hearts were sampled by an expert pathologist, formalin fixed, and paraffin embedded. Immunohistochemistry was carried out using the following antibodies: anti-ghrelin Ab (antibodies-online.com, Aachen, Germany; Rabbit polyclonal antibody, dilution 1:100 v/v, 40’ at RT), anti-GHSR (Abcam, Cambridge, UK, Rabbit polyclonal antibody, dilution 1:1200 v/v, 40’ at RT), and anti-IL-1β (Abcam, Rabbit polyclonal antibody, dilution 1:50 v/v, overnight at 4 °C). Antigen retrieval was conducted with citrate buffer, pH6 for both ghrelin and GHSR and Tris-EDTA buffer, and pH9 for IL-1β. Reactions were detected with Envision Kit (Agilent Dako, Santa Clara, CA, USA) using a peroxidase-conjugated polymer, which carries antibodies to rabbit and mouse immunoglobulins and diaminobenzidine as chromogen substrate. Sections were counterstained with Mayer Hematoxylin. Pictures were captured with a Leica DMD108 (Leica Microsystems, Wetzlar, Germany) microscope. In order to obtain a semiquantitative assessment of immunohistochemistry, some methodological precautions were taken. Specifically, for each antigen, all cardiac sections were stained during the same experimental session and images were taken keeping constant illumination and acquisition parameters. Concerning image analysis (that was carried out employing ImageJ software [15]), each image was first split into RGB channels. The channel with the highest signal to noise ratio (Red) was then used to analyze the positivity. In order to get the highest results with the highest positivity, the image was first inversed. Subsequently, a threshold was applied and both the average intensity of grey values of the positive signal and the area covered by positivity were measured. Integrated optical densities were finally computed multiplying the positive area by the average grey value of the positive signal.

### 2.4. Statistical Analysis

Characteristics of the study population are described using means ± SD or median and interquartile range for continuous variables, depending on the distribution’s shape, and percentages for categorical variables. Data were tested for normal distribution using the Kolmogorov–Smirnov test. *T*-test or Mann–Whitney test, as appropriate, were used to compare continuous variables between two groups. For categorical variables, cross-tabulations were generated, and chi-square or Fisher exact test was used to compare distributions. Correlations between variables with normal distribution were expressed with “r” of Pearson coefficient, while Rho of Spearman was used for non-Gaussian variables. 

The distribution of biomarkers was highly skewed and was therefore log-transformed prior to regression analyses.

By means of linear regression analyses we modeled the effect of DCM duration, left ventricle’s (LV) function, NYHA class, biomarker levels, gender, and age as predictors of ghrelin levels on the natural logarithm scale.

To evaluate time-to-event (death or cardiac transplantation) predictors the Cox regression was used, by estimating univariable and multivariable models. A stepwise algorithm “Backward Conditional” was used to select the sub-groups of independent factors at the multivariable analysis. The confidence interval (CI) was set at 95%.

Analyses were conducted with IBM-SPSS 21 statistical software and the R package version 3.2.2, using the “Matching” library to match DCM cases with healthy controls on age, sex and BMI [16]. All values with *p* < 0.05 were considered significant.

## 3. Results

### 3.1. Study Population: Comparison Controls vs. Patients with DCM

In the study we included 466 individuals (mean age 53 (±13), median 55, range 46–63 years, 79.3% male gender); 266 patients affected by DCM and 200 healthy controls, matched for age, gender and body mass index. Table 1 summarizes the available data for the two groups.

Patients affected by DCM had significantly lower levels of total, acylated, unacylated ghrelin when compared to matched healthy subjects (*p* < 0.001 for all the forms). Further, acylated/unacylated and acylated/total ghrelin ratios were higher in control subjects in comparison to patients with DCM (Table 1). In healthy controls, we observed a negative correlation of ghrelin with age, male gender and BMI (Table 2). Gender differences in ghrelin levels were also present in the DCM affected group, but no differences in ghrelin levels were seen with age or BMI (Table 2). 

### 3.2. Ghrelin in Patients with DCM 

The patient cohort comprised 266 outpatients with DCM (mean age of 54.5 years, 77.8% males, 73% in NYHA class I-II, see Table 3). Ejection fraction averaged 34.8% with mean LV end-diastolic volume index of 89 mL/m^2^ and average E/e’ ratio of 10.7. Left bundle branch block was present in a third of the population. 

### 3.3. Ghrelin and DCM Duration

The median duration of myocardial disease was 62.8 months (IQR: 15.4–155.8 months). Since we were interested in the effect of the duration of disease on ghrelin levels, we divided the patients according to DCM duration into three groups (less than 12 months, between 12–60 months and over 60 months) (Table 3). In patients with a more recent diagnosis of DCM, total ghrelin was higher (<12 months vs. 12–60 months vs. >60 months: 617 pg/mL (IQR: 322–983) vs. 461 pg/mL (IQR: 231–841) vs. 407 pg/mL (IQR: 292–693), respectively, *p* = 0.033). When compared to patients with longer DCM duration, we observed a trend towards increased unacylated ghrelin levels in the more recently diagnosed patients (*p* = 0.075). Last, acylated ghrelin levels did not differ among the three groups (*p* = 0.39). However, when we computed the acylated/unacylated ghrelin ratio and the acylated/total ghrelin ratio, we observed that they were significantly lower in patients with a more recent diagnosis. There were no differences in gender (*p* = 0.8) and BMI (*p* = 0.13) between the three groups with different length of DCM. 

### 3.4. Ghrelin and LVEF 

Next, we stratified patients with DCM in three groups according to the left ventricular ejection fraction (Table 4). As expected, the majority of patients (174 out of 266) had LVEF under 40% (median LVEF 29% (IQR 24–34%)), 61 patients had LVEF 40–49% (median LVEF 44 % (IQR 41–46%)) and 30 patients had LVEF > 50% (median LVEF 55% (IQR 51–59%). Patients with LVEF > 50% belonged to the category of “apparent healing” DCM [17]. There was no difference in duration of myocardial disease (*p* = 0.28), BMI (*p* = 0.13) and gender (*p* = 0.48) among the three groups with different LVEF.

Total ghrelin was higher in patients with lower left ventricular systolic function (<40% LVEF, vs. LVEF between 40% and 49% vs. LVEF ≥ 50%: 480.8 pg/mL (IQR: 306.2–855.2), vs. 429.7 pg/mL (IQR: 293–807) vs. 329.5 pg/mL (IQR: 223.2–557.6) respectively, *p* = 0.05), (Table 4). Additionally, in patients with reduced left ventricular systolic function, a trend towards higher levels of unacylated ghrelin was observed (*p* = 0.076). No differences were observed regarding acylated ghrelin levels among the three groups with different LVEF. 

Total ghrelin, acylated and unacylated ghrelin levels did not differ statistically among patients in NYHA I/II compared against class III/IV (*p* = 0.75, *p* = 0.56 and *p* = 0.92, respectively).

### 3.5. Correlations Analyses

We next evaluated correlations among serum biomarkers and clinical and instrumental parameters in patients with DCM; all ghrelin forms were positively correlated with BNP and sST2. Acylated ghrelin positively correlated also with IL-1β, and negatively with left ventricular mass. Total and non-acylated ghrelin positively correlated with IL-, while there was a trend suggesting a negative correlation with left ventricular ejection fraction. Other correlations between tested biomarkers and clinical-instrumental parameters are shown in Table 5. 

### 3.6. Predictors of Ghrelin Levels 

Predictors of Ln (total ghrelin) levels at multivariable backward stepwise linear regression analysis were LVEF (β = −0.7, *p* = 0.01), lnIL-1β levels (β = 0.2, *p* = 0.019) and lnIL-6 levels (β = 0.26, *p* = 0.003). Predictor of acylated ghrelin levels at multivariable analysis was lnIL-1β (β = 0.12, *p* = 0.007). 

### 3.7. Outcome Analyses

During the median follow-up of 56 months (IQR: 15.6–114.3 months), 40 patients died or underwent heart transplantation. Table 6 depicts baseline clinical characteristics of patients stratified by the end-point death/heart transplantation during the long-term follow-up. Patients who died/underwent heart transplantation had significantly higher levels of total ghrelin levels, BNP, sST2, galectin-3 and IL-6 when compared with patients without clinical events (Table 6).

At the Cox univariable and multivariable analyses, none of the ghrelin forms were independently associated with the outcome; advanced NYHA class, left ventricular ejection fraction and BNP levels were independent predictors of mortality/heart transplantation during the follow-up (Table 7).

### 3.8. Histological Analysis

In samples obtained from explanted, failing and normal hearts (whose clinical and anatomical characteristics are shown in Table 8), we evaluated the expression of ghrelin prepropeptide, the ghrelin receptor growth hormone secretagogue-receptor (GHSR), and IL-1β. 

**Figure 1:**
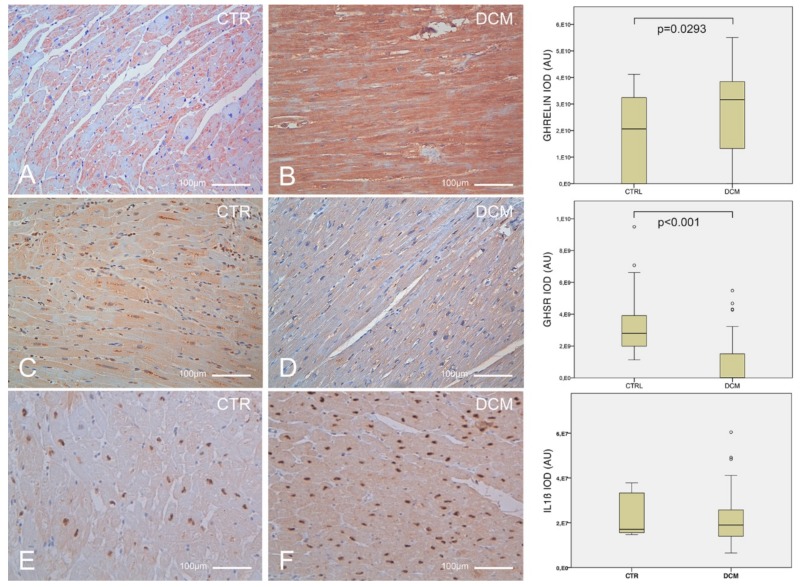
Quantitative assessment of ghrelin, leptin and of their receptors in human cardiac biopsies. Identification by immunohistochemistry staining (brown) of ghrelin prepropeptide (**A**,**B**) growth hormone secretagogue-receptor (**C**,**D**), and interleukin-1β (**E**,**F**) expression in tissue biopsies of control hearts (**A**,**C**,**E**) and hearts explanted from patients affected by dilated cardiomyopathy (**B**,**D**,**F**). Scale bars = 100 µm. Boxplots summarize the results of the quantitative assessment of each staining, evaluated as integrated optical density (IOD).

Ghrelin prepropeptide was expressed more in DCM patients than in healthy controls (*p* = 0.0293) while GHSR had an opposite trend of expression (*p* < 0.001). Intriguingly, IL-1β did not reach statistical significance. Upon examination of the correlation among the analyzed parameters, ghrelin showed an inverse correlation with its receptor (ρ = −0.406, *p* = 0.009, *n* = 41), while IL-1β inversely correlated with GHSR (ρ = −0.422, *p* = 0.0103, *n* = 36) (Figure 1).

## 4. Discussion 

In this work, we thoroughly investigated, for the first time, the association between the levels of ghrelin, myocardial disease duration, left ventricular ejection fraction and NYHA class in a large cohort of patients with DCM and long-term follow-up. Also, in a broader cohort to date, we evaluated the expression of ghrelin and GHS-receptor in the myocardium of patients with DCM and in controls.

Although ghrelin is a hormone that has been mainly studied for its involvement in the regulation of appetite, food intake, and energy expenditure, it has been shown to play a key role in regulating immunity, blood pressure, as well as insulin resistance, metabolic syndrome and cardiovascular disease [18]. Ghrelin is a growth hormone secretagogue (GHS) that promotes the release of growth hormone (GH) by binding to specific receptor subtypes present in the pituitary gland and in the hypothalamus. Intriguingly, GHS receptors have been identified in human cardiovascular tissue at higher density than in the hypothalamic/pituitary system [19]. Of note, ghrelin is a 28 amino acid peptide that derives from the maturation of a prepropeptide of 117 residues. Intriguingly, it was shown that a 23 amino acid peptide named obestatin derives from the same precursor [6]. Although obestatin exerts opposing effects with respect to ghrelin on food intake, it protects the heart from ischemia reperfusion injury by modulating the nitric oxide/protein kinase G pathway [9,20]. 

In physiological conditions, the majority of the circulating ghrelin is produced by the stomach and the small intestine [21], and much smaller quantities are produced by other tissues and organs such as cardiomyocytes [22].

In our study, we have observed that the circulating levels of every isoform of ghrelin is reduced in DCM patients compared to healthy controls matched for gender, age and BMI. Similar data were observed, in our previous work, in the setting of patients with acute coronary syndrome [18]. Consistent with our data, Chen et al. found lower levels of ghrelin in patients with chronic heart failure from ischemic–hypertensive-valvular origin compared to healthy controls [23].

Plasma ghrelin levels are influenced by age, gender, and BMI [24], and in our healthy controls we observed a similar negative correlation with age, male gender and BMI. Conversely, in patients with DCM, BMI and age were no longer correlated with reduced ghrelin levels. Furthermore, patients with a more recent diagnosis of DCM had higher ghrelin levels when compared to patients with longer duration of the DCM. This finding may be explained in light of both: the ability of BNP to inhibit the fasting-induced increase in acylated and unacylated ghrelin in healthy men [25], and of the possible role of inflammation in inducing cachexia and possibly suppressing the ghrelin axis [26]. 

Further, we also observed a seemingly contrasting finding; at all timeframes after diagnosis of DCM, patients with lower LVEF showed higher ghrelin levels. Also, at multivariable regression analysis, lower LVEF was associated with higher ghrelin levels. The observed increase in ghrelin levels could be a compensatory reaction to the worsening of the left ventricular function and the consequent lower cardiac output. Indeed, ghrelin resistance has been observed to occur in end-stage heart failure and to be reversed after cardiac transplantation [27].

Exploring the effect of ghrelin on the cardiovascular system, Nagaya et al. demonstrated that ghrelin administration reduces cardiac afterload and increases cardiac output both in healthy volunteers [28] and in patients with heart failure [29]. Furthermore, the exogenous administration of ghrelin reduces the norepinephrine plasma levels and increases the ejection fraction of patients with heart failure [30]. Importantly, the positive effect of the ghrelin analog hexarelin is not apparent in patients affected by dilated cardiomyopathy [31,32]. Imazio et al. [31] observed that in patients with DCM and severe left ventricular dysfunction, acute hexarelin administration did not improve LVEF, as opposed to patients with severe left ventricular dysfunction of ischemic origin. This finding may be partially explained by the observed reduction of GHSR in explanted hearts from our patients with DCM, as well as what was previously found in models of diabetic cardiomyopathy [33]. Conversely, Beiras-Fenandez et al. [34] in a study including 12 patients with end-stage heart failure (whose etiology was not specified), undergoing heart transplantation, observed an increase in myocardial GHSR1a expression in comparison to three cardiac autopsy tissue controls. Similarly, Sullivan et al. [35] in a tissue samples from 10 patients (5 of which with ischemic heart disease) undergoing heart transplantation observed an increase in GHSR in comparison to the myocardial biopsies of the grafted heart during the follow-up. Thus, the difference in the response to ghrelin administration in heart failure due to distinct etiologies [31,36] may be due to the different expression patterns of ghrelin and its receptor. However, we do not know when, in the natural history of patients with DCM, the downregulation of ghrelin receptor occurs. Recently, Sullivan et al. [33] examined changes of ghrelin receptor GHSR1a along with variations of left ventricular function in mouse models of diabetic cardiomyopathy; they observed that the decrease in myocardial expression of GHSR1a occurs already in the early phase of the disease, even before fibrosis development. Therefore, we could speculate that the increased ghrelin levels (both in plasma and tissue) may be a compensatory mechanism for the reduced tissue sensitivity to the hormone. However, in the setting of downregulation of receptors, patients with DCM cannot benefit from the protective effect of ghrelin. Sullivan and collaborators hypothesize that alteration in expression in ghrelin receptor observed in diabetic cardiomyopathy could be a consequence of upregulation of inflammatory factors such as IL-1β, IL-6 [33]. Their hypothesis is in line with our observation of a negative correlation between GHSR1 and IL-1β in patients with DCM. Intriguingly, Lund et al. observed that ghrelin resistance observed in patients with severe heart failure, caused by different etiologies, resolves after heart transplantation [27].

When assessing the association of ghrelin with other clinical and humoral parameters, we observed that all ghrelin forms were positively correlated with the markers of heart failure associated with cardiac stress, fibrosis and remodeling such as BNP and sST2. Nevertheless, ghrelin levels were not independently associated with mortality/cardiac transplantation during long-term follow-up. Chen et al. also observed a positive correlation between ghrelin and NT-pro BNP; further, in their study low ghrelin levels were associated with increased risk of adverse cardiac events during two-year follow-up. This difference in results is possibly due to the difference in the population included (age, etiology of HF, ghrelin levels) and the duration of the follow-up. Patients included in our study were younger, had HF due to DCM, had considerably higher levels of ghrelin and longer-term follow-up.

Study limitations: we did not perform bioelectrical impedance analysis (BIA) to assess body composition. However, none of included patients was obviously cachectic and the lowest BMI was above 18 kg/m^2^. Since Ghrelin is a GH secretagogue, it would have been interesting to dose the levels of this hormone in the plasma of enrolled subjects and to correlate them with Ghrelin levels. For practical reasons we did not have the chance to do it in the present work, therefore additional works should specifically address this issue. Although the number of samples for histological analyses may be considered limited, this is one of the largest studies present in the literature, given that endomyocardial biopsies and cardiac transplantations are conducted in few, very specialized centers [37,38]. 

In summary, our data suggest that DCM duration and severity is accompanied by alterations in the ghrelin–GHSR system. The decreased ghrelin levels following the diagnosis of DCM are consistent with previous studies on HF of different etiology [23]. Ghrelin has a favorable effect on cardiovascular function and has been considered as a potentially therapeutic hormone [39]. Further studies should address factors associated with the response to ghrelin such as to directly assess how the etiology of heart failure impacts on the downregulation of GHSR and to identify if inflammation or other factors may modulate GHSR expression and ghrelin sensitivity, in a way to reverse ghrelin resistance and to restore its protective effects on the myocardium.

## Figures and Tables

**Table 1:** Baseline characteristics of healthy subjects and patients affected by dilated cardiomyopathy (DCM).

	Healthy *n* = 200	DCM *n* = 266	*p*
**Age (years)**	52.4 (11.7)	54.5 (14)	0.074
**Male gender (%)**	80.8	77.7	0.4
**BMI (kg/m^2^)**	26.8 (5.15)	26.7 (4.6)	0.7
**Systolic blood pressure (mmHg)**	141.6 (16.6)	119.9 (15.9)	<0.001
**Diastolic blood pressure (mmHg)**	84.8 (10.8)	76 (12)	<0.001
**Total ghrelin (pg/mL)**	741 (481–1205.7)	461 (293.7–798.7)	<0.001
**Acylated ghrelin (pg/mL)**	51.8 (35.5–127.1)	21.7 (15.9–29.4)	<0.001
**Unacylated ghrelin (pg/mL)**	608.5 (443.3–1016.7)	435.4 (263.8–769.3)	<0.001
**Acylated/unacylated ghrelin**	0.09 (0.059–0.186)	0.05 (0.027–0.089)	<0.001
**Acylated/total ghrelin**	0.083 (0.056–0.16)	0.05 (0.03–0.08)	<0.001

Legend: BMI: body mass index.

**Table 2:** Correlations in healthy controls and in patients with DCM.

		Healthy Controls			DCM		
		Age	Male Gender	BMI	Age	Male Gender	BMI
**Total ghrelin**	ρ *p*	−0.355 <0.001	−0.239 <0.001	−0.570 <0.001	0.107 0.08	−0.126 0.04	0.04 0.52
**Acylated ghrelin**	ρ *p*	−0.288 <0.001	−0.152 0.01	−0.494 <0.001	0.108 0.08	−0.142 0.02	−0.037 0.55
**Unacylated ghrelin**	ρ *p*	−0.371 <0.001	−0.253 <0.001	−0.551 <0.001	0.102 0.1	−0.126 0.04	0.039 0.53

Legend: DCM: dilated cardiomyopathy.

**Table 3:** Main characteristics of patients stratified by DCM duration.

	All Patients *n* = 266 (100%)	DCM ≤ 12 Months *n* = 58 (21.8%)	12 < DCM ≤ 60 Months *n* = 72 (27.1%)	DCM > 60 Months *n* = 136 (51.1%)	*p*
**Age (years)**	54.5 (14.5)	49.5 (14.8)	51.47 (15.7)	57.8 (12.9)	0.001
**Male gender (%)**	77.7	79.3	75.4	78.7	0.8
**BMI (kg/m^2^)**	26.7 (4.6)	26.4 (4.7)	26.5 (4.9)	26.8 (4.4)	0.13
**Systolic Blood Pressure (mmHg)**	119.9 (15.9)	121.6 (16.6)	115 (16)	122 (15)	0.13
**Diastolic Blood Pressure (mmHg)**	76 (12)	77.5 (11)	76 (15)	76 (10)	0.59
**Heart Rate (bpm)**	69.9 (14.2)	73 (18)	70 (13)	69 (13)	0.18
**NYHA class I-II (%)**	73.5	67.2	72.5	77	0.35
**Sinus rhythm (%)**	86	93.1	89.9	81.6	0.20
**LBBB (%)**	38.5	32.8	37.7	40.4	0.6
**CRF (%)**	20.5	14	19.4	23.5	0.33
**Anemia (%)**	16.7	12.3	19.1	17.6	0.56
**Sodium (mEq/L)**	139.1 (2.7)	139 (2.8)	138.6 (3.3)	139 (2.3)	0.27
**Left atrial diameter indexed (cm/m^2^)**	21.9 (5.4)	22 (4.8)	20.7 (5)	22.5 (6)	0.10
**Left atrial area (cm^2^)**	25.3 (8.1)	14 (4)	12 (3.5)	13 (4)	0.09
**LVEDDI (cm/m^2^)**	32.3 (28.7–36)	33.7 (28.6–37.6)	30.7 (27.9–34.6)	33 (29–36.2)	0.088
**LVESDI (cm/m^2^)**	26 (22–30)	27.8 (21.8–30.8)	24.5 (20.1–28.1)	26.5 (22.6–30.8)	0.015
**IVS (cm)**	1.01 (0.2)	1 (0.2)	1 (0.2)	1 (0.2)	0.53
**LVEDVI (mL/m^2^)**	89 (66.6–112)	95.9 (74–126)	82 (65–103)	87 (66–106)	0.051
**LVESVI (mL/m^2^)**	60 (39–78)	72 (47–87)	50 (39–68)	57 (36–75)	0.031
**LVEF (%)**	34.8 (26.7–42.9)	32 (25–40)	36 (29–42)	36 (27–45)	0.07
**E/A ratio**	1.06 (0.75–1.6)	1.2 (0.8–2.2)	1.1 (0.8–1.5)	1 9(0.7–1.5)	0.24
**E/E’ ratio**	10.7 (8–14)	11 (8.7–14)	10 (8–13)	11 (8–14)	0.18
**WMSI**	1.96 (0.4)	2 (0.5)	1.9 (0.4)	1.9 (0.5)	0.52
**LV mass (g)**	261 (208–317)	257 (219–329)	254 (197–290)	262 (210–316)	0.4
**MR grade 2–4 (%)**	47.1	47.4	42.6	48.9	0.7
**Total ghrelin (pg/mL)**	461 (293.7–798.7)	617 (322–983)	461 (231–841)	407 (292–693)	0.033
**Acylated ghrelin (pg/mL)**	21.7 (15.9–29.4)	20.4 (14.8–27.5)	22.6 (17–32)	21.7 (17–30)	0.39
**Unacylated ghrelin (pg/mL)**	435.4 (263.8–769.3)	583 (294.3–897.6)	438.2 (199.8–831)	384.3 (273.5–663.7)	0.075
**Acylated/unacylated ghrelin**	0.05 (0.0269–0.088)	0.0326 (0.02–0.065)	0.046 (0.017–0.11)	0.0568 (0.033–0.097)	0.019
**Acylated /total ghrelin**	0.049 (0.0267–0.081)	0.0316 (0.02–0.061)	0.0489 (0.02–0.1)	0.0542 (0.032–0.089)	0.013
**BNP (pg/mL)**	156 (68.9–256.4)	123.6 (44.5–229.9)	145 (78.6–243.3)	168.1 (83.4–307.2)	0.056
**sST2 (ng/mL)**	31.1 (17.9–62.1)	30.9 (17.9–63.5)	34 (20.6–60.7)	28.9 (16–62.9)	0.39
**Galectin-3 (ng/mL)**	9.08 (7–13.3)	9.15 (6.7–11.7)	8.6 (6.3–11.7)	9.3 (7.5–15.3)	0.07
**IL-1β (pg/mL)**	0.6 (0.08–1.5)	0.47 (0.06–1.55)	0.038 (0.07–1.2)	0.8 (0.09–1.67)	0.28
**IL-6 (pg/mL)**	1.5 (0.6–3.3)	1.28 (0.47–2.5)	1.6 (0.49–4.01)	1.67 (0.83–3.72)	0.048
**IL-10 (pg/mL)**	1.4 (0.5–5.3)	1 (0.14–6.76)	1.22 (0.52–5.47)	1.83 (0.56–4.89)	0.52
**TNF-α (pg/mL)**	11.1 (8.6–16)	10.3 (8.4–15.6)	10.3 (7.7–14.1)	11.7 (9.7–17.9)	0.06
**ACE-I/ARBs (%)**	92.1	93.1	89.9	92.6	0.74
**Beta-blockers (%)**	90.2	87.9	91.2	90.4	0.8
**Digitalis (%)**	24.9	8.6	10.1	39.7	<0.001
**Antiplatelets (%)**	27.5	22.4	27.5	29.4	0.61
**Oral Anticoagulants (%)**	26	22.4	16	33.1	0.023
**Amiodarone (%)**	17.4	19	13	19.1	0.53
**Diuretics (%)**	67.9	69	61	71	0.31
**Statins (%)**	29.5	20.4	27.5	34.6	0.19
**Oral antidiabetics (%)**	7.7	4.1	5.9	10.3	0.34
**ICD (%)**	25.1	12.2	27.5	29.9	0.06

Legend: NYHA: New York Heart Association; CRF: chronic renal failure; LBBB: left bundle branch block; LVEDDI: left ventricular end-diastolic diameter indexed; LVESDI: left ventricular end-systolic diameter indexed; IVS: intraventricular septum; LVEDVI: left ventricular end-diastolic volume indexed; LVESVI: left ventricular end-systolic volume indexed; LVEF: left ventricular ejection fraction; WMSI: wall motion score index; LV: left ventricular; MR: mitral regurgitation; BNP: brain natriuretic peptide; sST2: soluble suppressor of tumorigenicity-2; IL: interleukin; TNF-α: tumor necrosis factor alpha; ACE-I/ARBs: angiotensin converting enzyme inhibitors/angiotensin receptor blockers; ICD: implantable cardioverter–defibrillator.

**Table 4:** Main characteristics of patients stratified by LVEF.

	All Patients *n* = 266 (100%)	LVEF < 40% *n* = 174 (66%)	LVEF 40–49% *n* = 61 (23%)	LVEF ≥ 50% *n* = 30 (11%)	*p*
**Age (years)**	54.5 (14.5)	56.1 (13.7)	53.9 (15.7)	45.9 (14.2)	0.001
**Male gender (%)**	77.7	79.3	72.1	80	0.48
**BMI (kg/m^2^)**	26.7 (4.6)	26.9 (4.8)	25.6 (3.9)	26.9 (4)	0.13
**Overweight (%)**	60	63.8	47.5	63.3	0.08
**Obese (%)**	21.1	22.4	14.8	26.7	0.3
**Systolic blood pressure (mmHg)**	119.9 (15.9)	118.4 (15.8)	122.7 (17.2)	123.2 (12.9)	0.1
**Diastolic blood pressure (mmHg)**	76 (12)	74.9 (9.8)	78 (17.9)	78.2 (6.4)	0.1
**Heart rate (bpm)**	69.9 (14.2)	71.7 (15.5)	66.3 (11.2)	66.5 (9.6)	0.02
**NYHA class I-II (%)**	73.5	63.2	90.0	100.00	<0.001
**Sinus rhythm (%)**	86	81.6	91.8	100.0	0.012
**LBBB (%)**	38.5	43.7	37.7	10.0	0.002
**Diabetes mellitus (%)**	9.8	10.9	8.2	6.7	0.7
**COPD (%)**	5.3	6.9	3.3	0	0.22
**Smoking (%)**	21.5	24.7	13.1	20	0.16
**GFR (mL/min/1.73m^2^)**	77.3 (61.3–93.9)	74.7 (60–91)	80.9 (61–102)	83.5 (71–102)	0.024
**CRF (%)**	20.5	22.7	20.7	7.1	0.2
**Anemia (%)**	16.7	22.4	3.3	10.7	0.002
**Haemoglobin (g/dL)**	13.9 (1.5)	13.7 (1.6)	14.2 (1.3)	14.2 (1.4)	0.02
**Sodium (mEq/L)**	139.1 (2.7)	138.7 (2.9)	139.8 (1.9)	139.9 (1.9)	0.005
**Creatinine (mg/dL)**	1.01 (0.87–1.2)	1.04 (0.9–1.3)	0.97 (0.8–1.12)	0.98 (0.85–1.04)	0.017
**Left atrial diameter indexed (mm/m^2^)**	21.9 (5.4)	23.1 (5.5)	19.9 (4.7)	19.3 (4.2)	<0.001
**Left atrial area (cm^2^)**	25.3 (8.1)	27.8 (8.6)	22.3 (6.4)	21 (4.8)	<0.001
**LVEDDI (cm/m^2^)**	32.3 (28.7–36)	34 (30–38)	31 (28–34)	29 (26–32)	<0.001
**LVESDI (cm/m^2^)**	26 (22–30)	28 (24–32)	24 (20–26)	20 (18–22)	<0.001
**IVS (cm)**	1.01 (0.2)	1 (0.18)	1 (0.16)	1.1 (0.17)	0.2
**LVEDVI (mL/m^2^)**	89 (66.6–112)	101 (85–121)	66 (55–79)	57 (47–62)	<0.001
**LVESVI (mL/m^2^)**	60 (39–78)	71 (59–90)	38 (30–44)	25 (18–29)	<0.001
**LVEF (%)**	34.8 (26.7–42.9)	29 (24–35)	44 (41–46)	56 (52–60)	<0.001
**E/A ratio**	1.06 (0.75–1.6)	1.1 (0.75–1.8)	1.02 (0.78–1.3)	1.2 (0.84–1.46)	0.6
**E/E’ ratio**	10.7 (8–14)	11.9 (8.9–16.7)	10 (8–13)	8.4 (6.9–11)	< 0.001
**WMSI**	1.96 (0.4)	2.2 (0.45)	1.85 (0.3)	1.3 (0.3)	< 0.001
**LV mass (g)**	261 (208–317)	284 (242–342)	234 (191–268)	226 (183–282)	< 0.001
**MR grade 2–4 (%)**	47.1	58.2	31.1	16.7	< 0.001
**Total ghrelin (pg/mL)**	461 (293.7–798.7)	480.8 (306.2–855.2)	429.7 (293–807)	329.5 (223.2–557.6)	0.05
**Acylated ghrelin (pg/mL)**	21.7 (15.9–29.4)	22.2 (15.5–29.9)	21.6 (16–29.1)	20.8 (17.1–28.7)	0.9
**Unacylated ghrelin (pg/mL)**	435.4 (263.8–769.3)	452.1 (274.7–833.8)	406.3 (230–744.8)	305.9 (200–544.7)	0.076
**Acylated/unacylated ghrelin**	0.05 (0.0269–0.088)	0.0465 (0.024–0.085)	0.0461 (0.025–0.95)	0.075 (0.035–0.094)	0.092
**Acylated /total ghrelin**	0.049 (0.0267–0.081)	0.04 (0.024–0.08)	0.048 (0.025–0.09)	0.069 (0.034–0.086)	0.11
**BNP (pg/mL)**	156 (68.9–256.4)	155.5 (67.3–243.2)	135.8 (79.4–341.6)	201.8 (76.5–271.8)	0.9
**sST2 (ng/mL)**	31.1 (17.9–62.1)	31.6 (18.6–63.3)	30.2 (16.9–61.8)	30 (15.7–49.1)	0.4
**Galectin-3 (ng/mL)**	9.08 (7–13.3)	9 (7–12.9)	10.8 (7.6–17.4)	9.1 (6.5–10.6)	0.07
**IL-1β (pg/mL)**	0.6 (0.08–1.5)	0.5 (0.08–1.39)	0.6 (0.08–1.7)	1.1 (0.16–2.17)	0.3
**IL-6 (pg/mL)**	1.5 (0.6–3.3)	1.7 (0.68–3.9)	1.51 (0.48–3.11)	0.99 (0.61–1.49)	0.03
**IL-10 (pg/mL)**	1.4 (0.5–5.3)	1.35 (0.47–5.69)	0.9 (0.53–3.68)	3.19 (1.31–7.52)	0.06
**TNF-α (pg/mL)**	11.1 (8.6–16)	11.5 (9.01–17.2)	10.3 (7.9–13.9)	12.05 (7.4–21.9)	0.11
**ACE-I/ARBs (%)**	92.1	94.8	91.8	76.7	0.003
**Beta-blockers (%)**	90.2	91.4	90.2	83.3	0.4
**Digitalis (%)**	24.9	23.6	24.6	33.3	0.5
**Antiplatelets (%)**	27.5	28.2	27.9	23.3	0.9
**Oral Anticoagulants (%)**	26	30.5	13.1	26.7	0.03
**Amiodarone (%)**	17.4	20.1	9.8	16.7	0.2
**Diuretics (%)**	67.9	75.9	52.5	53.3	0.001
**Statins (%)**	29.5	30.3	32.7	20	0.4
**Oral antidiabetics (%)**	7.7	9	5.5	6.7	0.7
**ICD (%)**	25.1	32	14.5	16.7	0.02

**Table 5:** Correlations between serum biomarkers and clinical-instrumental parameters in patients with DCM.

		Total Ghrelin	Acylated Ghrelin	Unacylated Ghrelin
**Age**	ρ *p*	0.1 0.1	0.11 0.08	0.10 0.09
**Male gender**	ρ *p*	−0.124 0.04	−0.14 0.02	−0.13 0.039
**BMI**	ρ *p*	0.033 0.6	−0.03 0.6	0.04 0.56
**NYHA class**	ρ *p*	0.1 0.09	0.12 0.08	0.07 0.28
**LBBB**	ρ *p*	0.04 0.5	0.007 0.9	0.036 0.56
**Atrial fibrillation**	ρ *p*	0.037 0.5	0.023 0.7	0.04 0.5
**Diabetes mellitus**	ρ *p*	−0.062 0.32	−0.003 0.96	−0.06 0.36
**Smoking**	ρ *p*	0.1 0.09	0.119 0.055	0.09 0.14
**GFR**	ρ *p*	−0.1 0.11	−0.119 0.057	−0.1 0.1
**LVEDDI**	ρ *p*	0.01 0.9	−0.02 0.7	0.01 0.86
**LVESDI**	ρ *p*	0.007 0.9	−0.023 0.7	−0.006 0.9
**LVEDVI**	ρ *p*	0.12 0.08	−0.007 0.91	0.08 0.2
**LVESVI**	ρ *p*	0.1 0.08	0.01 0.87	0.08 0.19
**LVEF**	ρ *p*	−0.09 0.13	−0.05 0.42	−0.09 0.16
**LV mass**	ρ *p*	0.04 0.57	−0.16 0.019	0.02 0.7
**Death/transplant**	ρ *p*	0.12 0.05	0.045 0.5	0.09 0.12
**BNP**	ρ *p*	0.11 0.08	0.131 0.03	0.12 0.055
**sST2**	ρ *p*	0.12 0.048	0.124 0.045	0.12 0.049
**Galectin-3**	ρ *p*	0.042 0.5	0.075 0.23	0.025 0.7
**IL-1β**	ρ *p*	−0.015 0.8	0.3 <0.001	−0.004 0.95
**IL-6**	ρ *p*	0.15 0.013	−0.015 0.81	0.015 0.018
**IL-10**	ρ *p*	0.096 0.12	0.013 0.41	0.11 0.07
**TNF-α**	ρ *p*	0.064 0.31	0.22 <0.001	0.05 0.38

**Table 6:** Main characteristics of patients stratified by the end-point death/heart transplantation during the follow-up.

	All Patients *n* = 266 (100%)	Death/Transpl *n* = 42 (16%)	Alive *n* = 224 (84%)	*p*
**Age (years)**	54.5 (14.5)	55.1 (15.2)	54.4 (14.4)	0.8
**Male gender (%)**	77.7	92.9	74.9	0.01
**BMI (kg/m^2^)**	26.7 (4.6)	26.9 (4.7)	26.6 (4.5)	0.75
**Overweight (%)**	60	66.7	58.7	0.2
**Obese (%)**	21.1	21.4	21.1	0.96
**Systolic blood pressure (mmHg)**	119.9 (15.9)	117.4 (14.2)	120.4 (16.2)	0.3
**Diastolic blood pressure (mmHg)**	76 (12)	74.9 (8.6)	76.3 (12.5)	0.5
**Heart rate (bpm)**	69.9 (14.2)	72.9 (11.2)	69.3 (14.6)	0.2
**NYHA class I-II (%)**	73.5	57.1	76.6	0.009
**Sinus rhythm (%)**	86	69	89.2	0.002
**LBBB (%)**	38.5	47.6	36.8	0.18
**Diabetes mellitus (%)**	9.8	16.7	8.5	0.1
**COPD (%)**	5.3	11.9	4	0.052
**Smoking (%)**	21.5	28.6	20.2	0.2
**GFR (mL/min/1.73 m^2^)**	77.3 (61.3–93.9)	68.9 (51.7–89.2)	77.5 (64.1–94.7)	0.045
**CRF (%)**	20.5	40.5	16.7	0.001
**Anemia (%)**	16.7	33.3	13.6	0.002
**Haemoglobin (g/dL)**	13.9 (1.5)	13.8 (1.5)	13.9 (1.6)	0.7
**Sodium (mEq/L)**	139.1 (2.7)	138.2 (2.9)	139.2 (2.6)	0.02
**Creatinine (mg/dL)**	1.01 (0.87–1.2)	1.1 (0.97–1.4)	1 (0.84–1.2)	0.001
**Left atrial diameter indexed (mm/m^2^)**	21.9 (5.4)	24.8 (5.6)	21.4 (5.2)	0.001
**Left atrial area (cm^2^)**	25.3 (8.1)	28.9 (10)	24.8 (7.7)	0.02
**LVEDDI (cm/m^2^)**	32.3 (28.7–36)	32.5 (27.9–37.6)	32.2 (28.7–35.97)	0.82
**LVESDI (cm/m^2^)**	26 (22–30)	28 (24.38–33.8)	25.34 (21.45–30)	0.012
**IVS (cm)**	1.01 (0.2)	1.02 (0.2)	1 (0.17)	0.7
**LVEDVI (mL/m^2^)**	89 (66.6–112)	93.3 (78.9–113.1)	88.1 (65.02–111.2)	0.066
**LVESVI (mL/m^2^)**	60 (39–78)	66.8 (49.9–78.3)	56 (36.7–77.7)	0.042
**LVEF (%)**	34.8 (26.7–42.9)	29.7 (23.1–39.3)	35.6 (27.5–44)	0.012
**E/A ratio**	1.06 (0.75–1.6)	1.48 (1–2.21)	1.02 (0.75–1.5)	0.02
**E/E’ ratio**	10.7 (8–14)	13 (9.4–18.4)	10.3 (8–13.5)	0.04
**WMSI**	1.96 (0.4)	2.14 (0.4)	1.93 (0.4)	0.03
**LV mass (g)**	261 (208–317)	285.5 (215.8–332.3)	256 (205.8–314.8)	0.13
**MR grade 2–4 (%)**	47.1	60	44.8	0.07
**Total ghrelin (pg/mL)**	461 (293.7–798.7)	517.4 (329.4–1059.6)	430.3 (273–777)	0.05
**Acylated ghrelin (pg/mL)**	21.7 (15.9–29.4)	22.8 (18.2–29.9)	21.6 (15.6–29.4)	0.46
**Unacylated ghrelin (pg/mL)**	435.4 (263.8–769.3)	462.5 (299.1–977.8)	406.3 (244.7–735.6)	0.12
**Acylated/unacylated ghrelin**	0.05 (0.0269–0.088)	0.047 (0.031–0.075)	0.053 (0.024–0.095)	0.5
**Acylated /total ghrelin**	0.049 (0.0267–0.081)	0.045 (0.03–0.07)	0.05 (0.026–0.089)	0.39
**BNP (pg/mL)**	156 (68.9–256.4)	262 (130–437.6)	135.4 (66.2–230.1)	<0.001
**sST2 (ng/mL)**	31.1 (17.9–62.1)	45 (26.3–102.6)	30.2 (17–57.6)	0.009
**Galectin-3 (ng/mL)**	9.08 (7–13.3)	10.5 (8.6–20.8)	9 (6.9–12.2)	0.01
**IL-1β (pg/mL)**	0.6 (0.08–1.5)	0.9 (0.3–1.8)	0.5 (0.1–1.4)	0.08
**IL-6 (pg/mL)**	1.5 (0.6–3.3)	3.28 (1.05–8.43)	1.49 (0.62–2.96)	0.002
**IL-10 (pg/mL)**	1.4 (0.5–5.3)	2.86 (0.97–11.2)	1.21 (0.5–4.14)	0.012
**TNF-α (pg/mL)**	11.1 (8.6–16)	12.3 (10.3–20.9)	10.8 (8.3–15.3)	0.028
**ACE-I/ARBs (%)**	92.1	95.2	91.5	0.4
**Beta-blockers (%)**	90.2	95.2	89.2	0.2
**Digitalis (%)**	24.9	38.1	22.4	0.03
**Antiplatelets (%)**	27.5	28.6	27.4	0.9
**Oral Anticoagulants (%)**	26	47.6	22	0.001
**Antiarrythmics (%)**	35.8	47.6	33.6	0.08
**Amiodarone (%)**	17.4	26.2	15.7	0.1
**Diuretics (%)**	67.9	83.3	65	0.02
**Statins (%)**	29.5	19.2	30.9	0.2
**Oral antidiabetics (%)**	7.7	15.4	6.6	0.2
**ICD (%)**	25.1	34.6	23.8	0.2

Legend: Transpl: transplantation; COPD: chronic obstructive pulmonary disease; GFR: glomerular filtration rate.

**Table 7:** Predictors of mortality/heart transplantation at multivariable Cox proportional hazards regression analysis.

	Univariable		Multivariable	
	HR (95% CI)	*p*	HR (95% CI)	*p*
**LnBNP**	1.51 (1.13–2.03)	0.005	1.6 (1.22–2.23)	0.001
**LVEF for 10% points increase**	0.96 (0.93–0.99)	0.003	0.7 (0.51–0.98)	0.04
**NYHA I-II vs. III-IV**	3.25 (1.75–6.02)	<0.001	2.47 (1.26–4.86)	0.009
**LnsST2**	1.47 (1.08–1.99)	0.013	-	-
**Sodium**	0.88 (0.8–0.97)	0.015	-	-
**LnGal-3**	1.16 (0.75–1.8)	0.5	-	-
**Ln-Total ghrelin**	1.3 (0.9–1.81)	0.17	-	-
**MDRD**	0.99 (0.97–1.005)	0.21	-	-
**LBBB**	1.5 (0.83–2.79)	0.17	-	-

Legend: Gal-3: Galectin-3; MDRD: modification of diet in renal disease.

**Table 8:** Demographic, echocardiographic, and anatomical features of patients whose hearts were analyzed histologically.

	**DCM** **(*n* = 46)**	**Controls** **(*n* = 18)**	**Normal Values**	***p***
**Age (Year)**	55.7 (41.9; 62.5)	41.5 (31.5; 51.5)	-	<0.01
**Sex (M/F)**	38/8	8/10	-	<0.01
**Duration of disease (Years)**	6.6 (3.1; 12.3)	-	-	
**NYHA class (%)**				
**II**	9	-	-	-
**III**	67	-	-	-
**IV**	24	-	-	-
Echocardiography *				
**Left ventricular diameter (mm)**				
**Systolic**	66 (56; 72)	-	21.6–34.8	-
**Diastolic**	74 (67; 83)	-	37.8–52.2	-
**Left ventricular volumes (mL)**				
**End-diastolic**	183.5 (163.8; 262.3)	-	46–106	-
**End-systolic**	132.5 (120.0; 168.8)	-	14–42	-
**Left ventricular ejection fraction (%)**	23 (20; 27)	-	54–74	-
**Hemodynamics†**				
**Pulmonary artery pressure (mmHg)**				
**Systolic**	46 (31.5; 51.5)	-	15–25	-
**Diastolic**	24 (14.5; 29.5)	-	8–12	-
**Mean**	31 (20.5; 37.5)	-	10–20	-
**PCWP (mmHg)**	21 (15; 31)	-	6–12	-
**CI (Lmin^−1^m^−2^)**	2.3 (1.8; 2.7)	-	2.5–4.0	-
**Gross Anatomy ‡**				
**Heart weight (g)**	465 (400; 561)	340 (235; 412)	196–516	<0.01
**Transverse diameter (mm)**	130 (120; 140)	102 (85; 122)	-	
**Inner longitudinal diameter (mm)**	95 (85; 105)	71 (6; 77)	-	<0.01
**Wall thickness (mm)**				<0.01
**LV**	10 (8; 11.5)	14 (12; 14)	-	0.017
**RV**	3 (3; 5)	3 (2; 5)	-	N.S.
**Septum**	1 (1; 1.35)		-	N.S.

Normal values as in (*) Lang RM, Badano LP, Mor-Avi V, Afilalo J, Armstrong A, Ernande L, Flachskampf FA, Foster E, Goldstein SA, Kuznetsova T, Lancellotti P, Muraru D, Picard MH, Rietzschel ER, Rudski L, Spencer KT, Tsang W and Voigt JU. Recommendations for cardiac chamber quantification by echocardiography in adults: an update from the American Society of Echocardiography and the European Association of Cardiovascular Imaging. *Eur. Heart J. Cardiovasc. Imaging*. 2015; 16: 233–70; (†) Bangalore S and Bhatt DL. Images in cardiovascular medicine. Right heart catheterization, coronary angiography, and percutaneous coronary intervention. *Circulation*. 2011; 124: e428–33; (**‡**) Sheppard M. Practical Cardiovascular Pathology, 2nd edition: Taylor & Francis; 2011. Legend: CI: cardiac index; PCWP: pulmonary capillary wedge pressure; RV: right ventricle.
